# Supplementary material for: Global hypo-methylation in a proportion of glioblastoma enriched for an astrocytic signature is associated with increased invasion and altered immune landscape
Source: eLife. 2022 Nov 22;11:e77335. doi: 10.7554/eLife.77335 (PMC9681209; doi:10.7554/eLife.77335)
Supplement: Figure 2—source data 1. [file elife-77335-fig2-data1.zip › Figure_2_source_data_1/Figure_2B/homerResults/motif3.similar.html]

motif3

## Information for motif3

C
G
A
T
A
C
T
G
A
G
T
C
G
A
C
T
A
G
T
C
G
A
T
C
G
A
T
C
G
C
A
T
A
C
T
G
A
G
C
T
A
G
C
T
A
G
C
T
  
Reverse Opposite:  

T
C
G
A
T
C
G
A
T
C
G
A
T
G
A
C
C
G
T
A
C
T
A
G
C
T
A
G
T
C
A
G
C
T
G
A
T
C
A
G
T
G
A
C
G
C
T
A
  

|  |  |
| --- | --- |
| p-value: | 1e-84 |
| log p-value: | -1.954e+02 |
| Information Content per bp: | 1.453 |
| Number of Target Sequences with motif | 104.0 |
| Percentage of Target Sequences with motif | 4.50% |
| Number of Background Sequences with motif | 1.8 |
| Percentage of Background Sequences with motif | 0.51% |
| Average Position of motif in Targets | 97.5 +/- 56.1bp |
| Average Position of motif in Background | 80.6 +/- 63.1bp |
| Strand Bias (log2 ratio + to - strand density) | 0.4 |
| Multiplicity (# of sites on avg that occur together) | 1.05 |
| Motif File: | file (matrix) reverse opposite |

### Similar de novo motifs found

|  |  |  |  |  |  |  |  |
| --- | --- | --- | --- | --- | --- | --- | --- |
| Rank | Match Score | Redundant Motif | P-value | log P-value | % of Targets | % of Background | Motif file |
| 1 | 0.636 | A T G C G T A C A C G T C A T G A T C G C G T A C T G A A T C G T C G A C A T G C T A G T C A G C T G A T C A G T G C A | 1e-53 | -123.417592 | 3.33% | 0.55% | motif file (matrix) |
| 2 | 0.775 | G C T A A G T C G T C A C T A G C T A G A T G C C G T A A C T G | 1e-37 | -85.514247 | 3.55% | 0.84% | motif file (matrix) |
| 3 | 0.640 | A T G C A G T C G C T A C T A G A C T G A C T G C T G A A T C G A T G C A G C T A T G C A G T C A G T C A C T G A C T G | 1e-21 | -49.962811 | 3.29% | 0.94% | motif file (matrix) |
